# Impact of degradable nanowires on long-term brain tissue responses

**DOI:** 10.1186/s12951-016-0216-7

**Published:** 2016-08-09

**Authors:** Lina Gällentoft, Lina M. E. Pettersson, Nils Danielsen, Jens Schouenborg, Christelle N. Prinz, Cecilia Eriksson Linsmeier

**Affiliations:** 1 Department of Experimental Medical Science, Medical Faculty, Neuronano Research Center (NRC), Lund University, Scheelevägen 2, 223 81 Lund, Sweden; 2 Division of Solid State Physics/NanoLund, Lund University, P.O. Box 118, 221 00 Lund, Sweden

**Keywords:** Nanowires, Biocompatibility, Neural interfaces, Brain, Biomaterial, Foreign body reaction, Tissue responses, Immunohistochemistry, Nanomedicine

## Abstract

**Background:**

A promising approach to improve the performance of neural implants consists of adding nanomaterials, such as nanowires, to the surface of the implant. Nanostructured interfaces could improve the integration and communication stability, partly through the reduction of the cell-to-electrode distance. However, the safety issues of implanted nanowires in the brain need to be evaluated and understood before nanowires can be used on the surface of implants for long periods of time. To this end we here investigate whether implanted degradable nanowires offer any advantage over non-degradable nanowires in a long-term in vivo study (1 year) with respect to brain tissue responses.

**Results:**

The tissue response after injection of degradable silicon oxide (SiOx)-coated gallium phosphide nanowires and biostable hafnium oxide-coated GaP nanowires into the rat striatum was compared. One year after nanowire injection, no significant difference in microglial or astrocytic response, as measured by staining for ED1 and glial fibrillary acidic protein, respectively, or in neuronal density, as measured by staining for NeuN, was found between degradable and biostable nanowires. Of the cells investigated, only microglia cells had engulfed the nanowires. The SiOx-coated nanowire residues were primarily seen in aggregated hypertrophic ED1-positive cells, possibly microglial cells that have fused to create multinucleated giant cells. Occasionally, degradable nanowires with an apparently intact shape were found inside single, small ED1-positive cells. The biostable nanowires were found intact in microglia cells of both phenotypes described.

**Conclusion:**

The present study shows that the degradable nanowires remain at least partly in the brain over long time periods, i.e. 1 year; however, no obvious bio-safety issues for this degradable nanomaterial could be detected.

**Electronic supplementary material:**

The online version of this article (doi:10.1186/s12951-016-0216-7) contains supplementary material, which is available to authorized users.

## Background

Micro- and nanostructured electrode surfaces have been suggested to improve recording properties and reduce tissue responses [1–7]. Thus, combining a nanostructured topography on a small, low-density and flexible interface, known to reduce glial scarring [8–10], opens up for the development of a new type of biocompatible neural interface, with potential to achieve high quality recordings from single neurons. One way of creating neural interfaces with nanostructured topography is to coat the electrode surface with nanowires [5, 11–15].

Cells have been shown to be able to grow and interact strongly with arrays of nanowires or nanopillars in vitro [13, 14, 16–20]. In particular, gallium phosphide (GaP) nanowire arrays have been shown to promote neurite outgrowth and reduce glial cell spreading [21, 22]. Previously, we have achieved successful acute in vivo recordings using neural interfaces with GaP nanowire surface modifications [15]. In order to test the biocompatibility of nanowires per se, we have recently investigated the brain tissue response to the injection of nanowires in the brain. In a first, relatively short-term nanosafety study, we used 2 µm long GaP nanowires coated with silicon oxide (SiOx) and assessed the tissue response 1, 6 and 12 weeks after injection [23]. One important finding of this study was that many, but not all, of the injected SiOx-coated nanowires had lost their structural integrity, i.e. both the GaP core and the SiOx coating were found to be degraded into fragments in vivo, within 12 weeks. The degraded nanowire fragments were found engulfed by macrophages/microglia in the injection tract [23], indicating that the nanowire material used could be fragmented or dissolved in the brain tissue but not cleared from the brain after 12 weeks.

However, it is unknown whether degradable nanowires will be completely removed from the injection site and eventually cleared from the brain over time. Importantly, it is also not known if degradable nanowires offer any advantage over non-degradable nanowires with regard to long-term inflammatory brain tissue response and neuronal survival.

The purpose of the present study was thus to evaluate how degradable nanowires affect the long-term inflammatory brain tissue response and neuronal density and to compare with the effects of implanted non-degradable (i.e. biostable [24]) nanowires, which are known to persist in the brain for long periods of time [25]. Further aims were to investigate the possible persistence of nanowires or nanowire residues in the brain tissue after 1 year and to clarify which of the different brain cells, such as glial and neuronal cells engulf the nanowires or their residues.

To this end, we evaluated the brain tissue response to 2 µm long degradable nanowires 1 year after injection. We compared the tissue response and neuronal survival in rat striatum after injections of degradable SiOx-coated GaP nanowires (2 µm) to biostable, hafnium oxide (HfOx)-coated GaP nanowires (2 µm), dispersed in Hank’s balanced salt solution (HBSS). One year post injection, the neuronal loss and the inflammatory tissue response were evaluated by quantification of microglia/macrophages, astrocytes, neuronal cell density, and total cell nuclei density, surrounding the nanowire injection site. The presence of nanowires and/or nanowire residues in cells and tissue was examined by detecting the scattered laser light in a confocal microscope.

## Methods

### Nanowire growth and coating

Metal organic vapor phase epitaxy (MOVPE) (Aix 200/4, Aixtron, Germany) was used to grow GaP nanowires from 40 nm gold aerosol particles on (111)B GaP substrates (Girmet Ltd, Moscow, Russia), as previously described [26]. The gold aerosol particles were randomly distributed at an average density of 1/µm^2^. The temperature for nanowire growth was 470 °C. The nanowire growth was initiated by supplying trimethylgallium (Ga(CH_3_)_3_) in addition to phosphine (PH_3_). The growth time was adjusted in order to obtain a nanowire length of 2 μm. Precursor molar fractions were 4.3 × 10^−6^ for Ga(CH_3_)_3_ and 8.5 × 10^−2^ for PH_3_. The hydrogen carrier gas flow was 6 L/min and the nanowire growth was conducted under low pressure (10 kPa).

The GaP nanowires were coated with a 20 nm layer of SiOx using Atomic layer deposition (Fiji, Cambridge NanoTech Inc., USA). The nanowires were subsequently broken off from the substrate using ultra sonication and suspended in HBSS as vehicle solution to a final concentration of 70,000 nanowires/µL.

The HfOx-coated nanowires were produced as described above except for the 20 nm HfOx atomic layer deposition, which was done using a Savannah-100 ALD system (Cambridge NanoTech Inc., USA), as published elsewhere [25].

### Animals

Approvals for the animal experiments were obtained in advance from the Lund/Malmö local ethical committee on animal experiments (ethical permit number: M300-10). Female Sprague–Dawley (SD) rats (Taconic, Denmark) were used. The rats were kept in a 12-h day-night cycle and received food and water ad libitum. At the beginning of the experiment the rats weighed approximately 225 g and they followed a normal weight curve post surgery.

At the experimental start-point, 10 animals received striatal bilateral injections of 2 µm long degradable SiOx-coated nanowires suspended in HBSS [rats = 10, n (injections) = 20], for details see below. Two of the rats were killed before the predetermined 1 year end point of the experiment, in accordance with the ethical exclusion criterion, since they developed age-related spontaneous tumors [25]. Furthermore, one rat was excluded since it did not meet our histological inclusion criteria. The results from the remaining seven rats (n = 14) were compared to data from our previously published study [25] where rats subjected to the same injection protocol, but injected with a suspension of 2 µm biostable HfOx-coated nanowires (n = 11), were investigated. The reason to compare the results from the present study with those from an already published study was ethical, and done in order to minimize the number of animals subjected to surgery and subsequently kept for a whole year after the nanowire injections.

### Surgery

The animals were anaesthetized by intraperitoneal (i.p.) injections of Fentanyl (0.3 mg/kg body weight) and Domitor vet (metedetomidin hydrochloride, 0.3 mg/kg body weight). The surgical area was shaved and the animal was positioned in a stereotactic frame (KOPF instruments, USA) under a stereomicroscope (Leica Microsystems, Germany). The scalp was disinfected using 70 % ethanol solution and local anaesthetic was administered, 0.25 % Marcaine (Bupivacaine, 0.33 mg/kg body weight) in sterile water. A 2 cm midline incision was made, connective tissue attached to the skull was carefully removed and blood was cleansed away. Under stereotactic control bilateral craniotomies (approximately ø 1 mm^2^) at 1.0 mm anterior and 2.5 mm lateral to bregma were drilled. Fine forceps were used to incise and deflect the dura mater, and stereotactic injections were made bilaterally using a 2 µL Hamilton syringe with a glass microcapillary (tip ø ~130 µm) attached. The HBSS suspension with nanowires was injected into the striatum at two depths; 5 mm (1 µL) and an additional injection at 4 mm (1 µL), i.e. 2 µL/hemisphere over a total time of 2 × 2 min.

The skin was closed with surgical clips. Before and after each session of nanowire injections, a drop of the nanowire suspension was ejected from the syringe onto a microscope slide and the presence of individually suspended nanowires was confirmed using a Nikon eclipse 80i microscope (Nikon, Japan).

After surgery, the animals were awakened under supervision. Subcutaneous injections of Temgesic (buprenorphine, 50 µg/kg body weight) were administered to reduce postoperative pain, as well as an antidote to the anaesthesia (Antisedan, atipamezole hydrochloride, 0.5 mg/kg body weight).

### Histology

The animals were killed by an i.p. overdose of pentobarbital 1 year post nanowire injection. The animals were transcardially perfused with ~200 mL of ice-cold saline solution (sodium chloride 0.9 % in distilled water) followed by ~125 mL of ice-cold 4 % paraformaldehyde (PFA) in 0.1 M phosphate buffered saline (PBS) (pH 7.4). The brains were gently removed and following post-fixation in 4 % PFA overnight (4 °C) they were cryo-protected in 25 % sucrose solution until equilibrated (4 °C). Cervical lymph nodes were also dissected and prepared for histology. The brains were snap frozen using dry ice and fixed to sectioning blocks using Tissue Tek optimal cutting temperature (O.C.T.) compound (Sakura Finetek, USA). Consecutive coronal sections were cut serially (6 series) at 10 µm thickness onto Super Frost^®^ plus slides (Menzel-Gläser, Germany) using a cryostat (Microm, Germany). The primary antibodies used to visualize activated microglia and macrophages [CD68 (ED1)], astrocytes [glial fibrillary acidic protein (GFAP)], and neuronal nuclei (NeuN) are summarized in Table 1. All stained sections were also counterstained using 4′,6-diamidino-2-phenylindole (DAPI), which labels all cell nuclei. The tissue sections were hydrated and rinsed three times using PBS and as blocking solution, 5 % normal goat serum in 0.25 % Triton X-100 (Fluka/Sigma-Aldrich, Switzerland) in PBS, was used. Following incubation with blocking solution (1 h), the first series of sections was stained with ED1 and GFAP. The second series was stained with NeuN and additionally co-stained with GFAP in order to visualize the scar. The sections were incubated with primary antibodies (in blocking solution) at room temperature (RT) overnight. The following day, sections were rinsed in PBS (three times) and incubated with DAPI, goat anti-mouse IgG Alexa 488 and goat anti-rabbit IgG Alexa 594 (in blocking solution), in light sealed chambers, for 2 h at RT (Table 1). Subsequently, sections were rinsed three times with PBS and coverslipped using PVA-DABCO (polyvinyl alcohol, Fluka/Sigma-Aldrich, Switzerland). For sections stained with NeuN (second series), an antigen retrieval protocol with a 10 mM sodium citrate buffer (0.05 % Tween 20, pH6) was performed, as previously described in Gällentoft et al. [25].

**Table 1:** List of primary antibodies, secondary antibodies and nucleic acid stain used in study

Name	Characteristics	Host	Working dilution	Source
ED1 (CD68)	Activated microglia/macrophages	Mouse	1:250	Cat. Nr. MCA341R, AbD Serotec, UK
GFAP	Glial fibrillary acidic protein	Rabbit	1:5000	Cat. Nr. Z0334, Dako, Denmark
NeuN	Neuronal nuclei, neuronal marker	Mouse	1:100	Cat. Nr. MAB377, Millipore, USA
Alexa Fluor 594	Goat anti-rabbit	Goat	1:500	Cat. Nr. A11005, Invitrogen, USA
Alexa Fluor 488	Goat anti-mouse	Goat	1:500	Cat. Nr. A11001, Invitrogen, USA
DAPI	Nucleic acid stain (4′,6-diamidino-2-phenylindole)	–	1:1000	Cat. Nr. D3571, Invitrogen, USA

The cervical lymph nodes were sectioned and labeled with ED1 and DAPI according to the same protocol.

### Image acquisition and analysis

A DS-Ri1 digital camera (Nikon, Japan) mounted on a Nikon eclipse 80i microscope (Nikon, Japan) with a 10× objective was used for image acquisition. The sections were screened for the scar using ED1- and GFAP-positive area. Where the scar was seen at its maximum, a photograph was taken of the fluorescence of the ED1- and GFAP-positive cells and cell nuclei (DAPI). These images were used for quantification of ED1, GFAP and DAPI. The adjacent brain sections in the second series were stained with the primary antibodies against NeuN and GFAP. These images were used for quantification of NeuN. A photograph of the fluorescence of GFAP-positive cells, NeuN-positive cells and cell nuclei (DAPI), was captured in the second series. The NIS-Elements 3.1 software (Nikon, Japan) was used for image capture and analysis. The quantification analysis was performed according to a previously described method [23, 25]. In short, a rectangular shaped region of interest (ROI; total ROI area 300 × 800 µm) was centred on the injection tract to evaluate the tissue response. This area was divided into an inner ROI (100 × 800 µm) and an outer ROI (200 × 800 µm). The inner ROI was chosen to quantify the area 0–50 µm from the injection tract and the outer ROI to quantify the area 50–150 µm from the injection tract. The rationale to differentiate between these two regions is that neuronal activity can be recorded up to about 50 µm from an electrode [27]. The outer ROI measures the possible widespread tissue response radiating from the injection scar. The quantifications were carried out by measuring the proportion of immunoreactive area (for ED1 and GFAP) or by counting the number of cells (for NeuN and DAPI) within the total screened area, i.e. inner or outer ROI [23, 25]. For ED1 and GFAP, intensity thresholds were set for each individual image and for each marker at a fixed multiplier of the mean background intensity. This was done in order to ensure that no unspecific staining was included in the area assigned as ED1 or GFAP-positive. Only the fraction of the area in the ROI above this set threshold was quantified. Intensity thresholds were set at 5.5 times the background intensity for ED1 immunofluorescence and at 4.5 times for GFAP immunofluorescence. Neuronal nuclei density was quantified manually by counting the number of NeuN-positive cells (with a DAPI-positive nucleus) and cell nuclei density were quantified by counting DAPI-positive nuclei (above ø 3 µm) within the ROIs.

In order to examine the presence (or absence) of nanowires in the tissue, we used confocal imaging. Confocal images of the scar were captured using a laser scanning confocal microscope (Zeiss LSM 510, Germany) with a 63× oil-immersion objective (N.A. 1.4) and Zen software (Zeiss, Germany). Laser-reflection mode was used to visualize the nanowires within the sections. Image J was used for processing of the confocal images.

The cervical lymph nodes were scanned for presence of nanowires or residues of nanowires using confocal microscopy (scattered laser light mode).

### Statistical analyses

SiOx-coated GaP nanowire injections, n = 14, were compared to HfOx-coated GaP nanowire injections, n = 11 (from a previous study [25]).

Mann–Whitney test was used for comparison of the two groups, P values <0.05 (*) were considered significant. Values within graphs are presented as median values with indication of the 25 and 75 percentiles and minimum and maximum values (boxplot). GraphPad Prism 6.0d software (GraphPad Software Inc., USA) was used to perform all analyses in the study.

The dataset supporting the conclusions of this article is included within the articles additional file (see Additional file 1).

## Results

To determine the impact of degradable vs. biostable nanowire exposure on the brain tissue response, we injected 2 µm long SiOx-coated GaP nanowires into rat striatum. This was compared to the tissue response after injection of HfOx-coated 2 µm GaP nanowires. The different immunohistochemical markers were quantified in an inner and an outer ROI surrounding the injection tract, 1 year post nanowire injection.

## Brain tissue response towards degradable vs. biostable nanowires after 1 year (inner ROI)

**Fig. 1:**
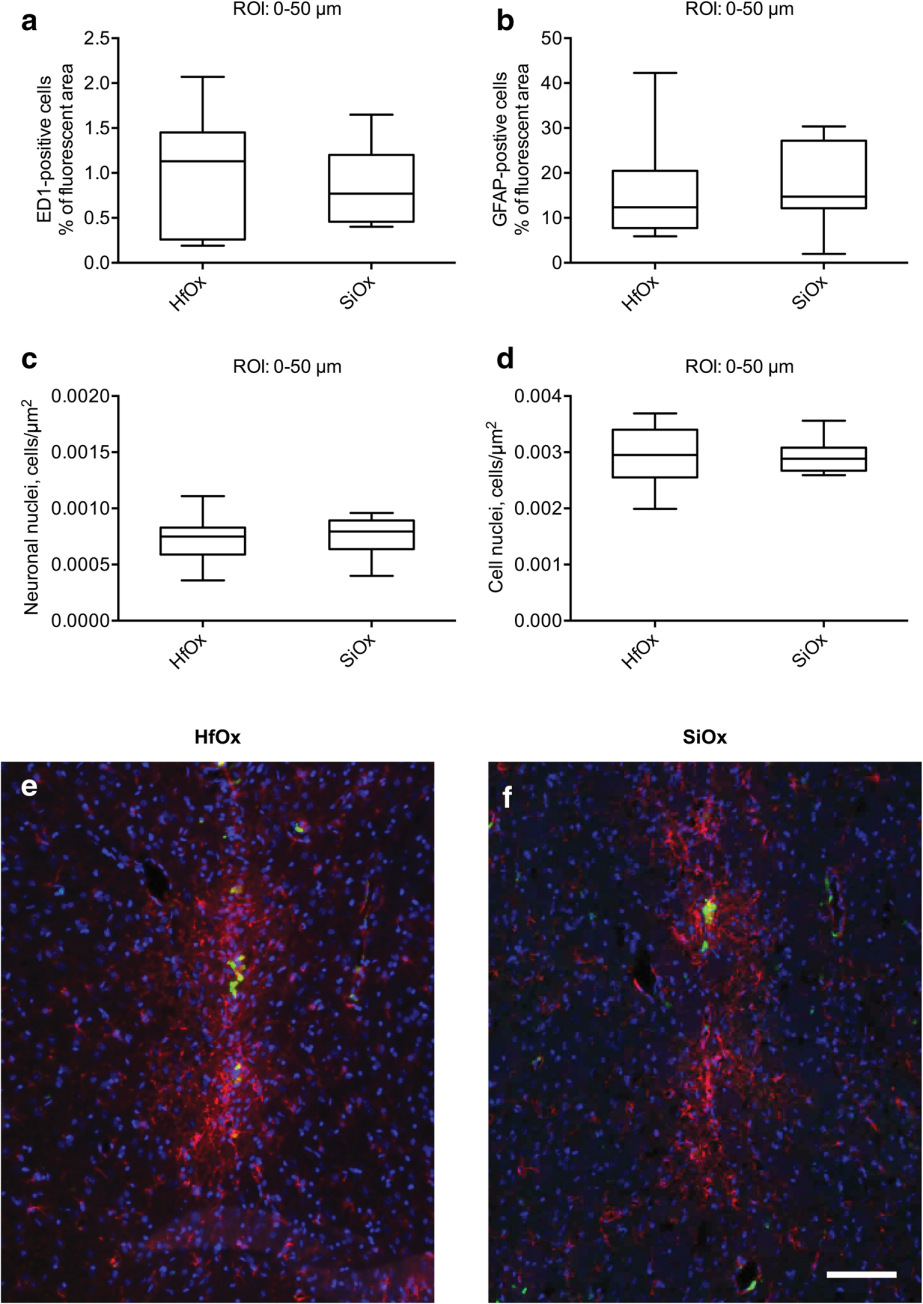
Inflammatory tissue response, cell nuclei and neuronal density (inner ROI). Quantification in the inner ROI (0–50 μm) of ED1-positive area (**a**), GFAP-positive area (**b**), NeuN density (**c**), and cell nuclei density (**d**) at 1 year for HfOx-coated GaP nanowires (biostable) and SiOx-coated GaP nanowires (degradable). The *boxes* correspond to median values with indication of the 25 and 75 percentiles, and the* whiskers* show the minimum and maximum values. Mann–Whitney test was used. Representative fluorescent images of the tissue response 1 year post injection of **e** 2 μm long HfOx-coated nanowires (biostable) and **f** 2 µm long SiOx-coated GaP nanowires (degradable). ED1-positive cells (*green*), GFAP-positive cells (*red*) and cell nuclei (*blue*). *Scale bar* 100 μm

One-year post injection no significant differences were found for any of the markers examined, when comparing the inner ROI (0–50 µm), in the two experimental groups. Boxplot graphs of the quantification in the inner ROI (a–d) and representative immunofluorescent images (e–f) are shown in Fig. 1.

For ED1, the median percentage of fluorescent area around the injection track of the SiOx-coated nanowires was 0.77 % (25 and 75 percentiles were 0.46 and 1.2 %, respectively), and 1.1 % (25 and 75 percentiles were 0.26 and 1.5 %, respectively) for the HfOx-coated nanowires (Fig. 1a).

The median percentage of GFAP-fluorescent brain area for animals injected with SiOx-coated nanowires was 15 % (25 and 75 percentiles were 12 and 27 %, respectively), and 12 % (25 and 75 percentiles were 7.7 and 20 %, respectively) for animals injected with HfOx-coated nanowires (Fig. 1b).

The median neuronal nuclei density, i.e. the number of neuronal nuclei (NeuN) per unit area in the inner ROI, was 8.0 × 10^−4^ µm^−2^ (25 and 75 percentiles were 6.4 × 10^−4^  and 8.9 × 10^−4^ µm^−2^, respectively) for SiOx-coated nanowires and 7.5 × 10^−4^ µm^−2^ (25 and 75 percentiles were 5.9 × 10^−4^ and 8.3 × 10^−4^ µm^−2^, respectively) for HfOx-coated nanowires (Fig. 1c).

The total cell nuclei density (DAPI), i.e. the number of cell nuclei per unit area in the inner ROI was 2.9 × 10^−3^ µm^−2^ (25 and 75 percentiles were 2.7 × 10^−3^ and 3.1 × 10^−3^ µm^−2^, respectively) for SiOx-coated nanowires and 3.0 × 10^−3^ µm^−2^ (25 and 75 percentiles were 2.6 × 10^−3^ and 3.4 × 10^−3^, respectively) for HfOx-coated nanowires (Fig. 1d).

## Tissue response towards degradable vs. biostable nanowires at 1 year (outer ROI)

**Fig. 2:**
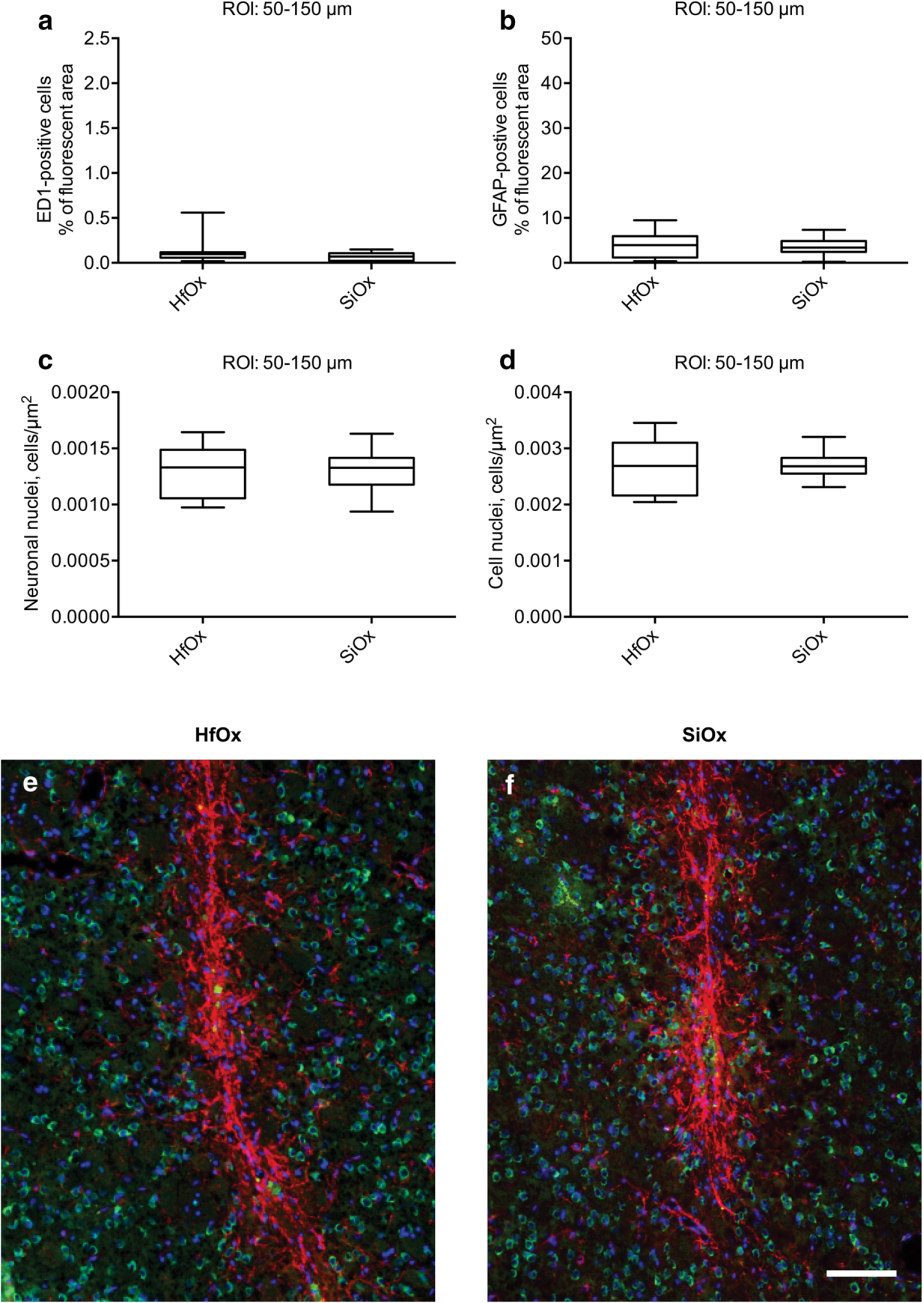
Inflammatory tissue response, cell nuclei and neuronal density (outer ROI). Quantification in the outer ROI (50–150 μm) of ED1-positive area (**a**), GFAP-positive area (**b**), NeuN density (**c**), and cell nuclei density (**d**) at 1 year for HfOx-coated GaP nanowires (biostable) and SiOx-coated GaP nanowires in vehicle solution (biostable). The *boxes* correspond to median values with indication of the 25 and 75 percentiles, and the* whiskers* show the minimum and maximum values. Mann–Whitney test was used. Representative fluorescent images of the tissue response 1 year post injection of **e** 2 μm long HfOx-coated nanowires (biostable) and **f** 2 µm long SiOx-coated GaP nanowires (degradable). Neuronal nuclei (*green*), GFAP-positive cells (*red*) and cell nuclei (*blue*). *Scale bar* 100 μm

One year after injection no significant differences were found, for any of the markers used, when comparing the outer ROIs (50–150 µm) of the two experimental groups. Boxplot graphs of the quantification in the outer ROI (a–d) and representative immunofluorescent images (e–f) are shown in Fig. 2.

For ED1 the median percentage of fluorescent area, in the outer ROI, was 0.069 % (25 and 75 percentiles were 0.022 and 0.11 %, respectively) for SiOx-coated nanowires and 0.098 (25 and 75 percentiles were 0.058 and 0.12 %, respectively) for HfOx-coated nanowires (Fig. 2a).

The median percentage of GFAP-fluorescent area was 3.4 % (25 and 75 percentiles were 2.4 and 4.9 %, respectively) for SiOx-coated nanowires and 3.9 % (25 and 75 percentiles were 1.2 and 5.9 %, respectively) for HfOx-coated nanowires (Fig. 2b).

The median neuronal nuclei density (NeuN) in the outer ROI, i.e. the number of neuronal nuclei per unit area was 1.3 × 10^−3^ µm^−2^ (25 and 75 percentiles were 1.2 × 10^−3^ and 1.4 × 10^−3^ µm^−2^, respectively) for SiOx-coated nanowires and 1.3 × 10^−3^ µm^−2^ (25 and 75 percentiles were 1.1 × 10^−3^ and 1.5 × 10^−3^ µm^−2^, respectively) for HfOx-coated nanowires (Fig. 2c).

The median nuclei density (DAPI) in the outer ROI, i.e. the number of cell nuclei per unit area, was 2.7 × 10^−3^ µm^−2^ (25 and 75 percentiles were 2.5 × 10^−3^ and 2.8 × 10^−3^ µm^−2^, respectively) for SiOx-coated nanowires, and 2.7 × 10^−3^ µm^−2^ (25 and 75 percentiles were 2.2 × 10^−3^ and 3.1 × 10^−3^ µm^−2^, respectively) for HfOx-coated nanowires (Fig. 2d).

### Confocal examination of the tissue response towards SiOx-coated (degradable) vs. HfOx-coated (biostable) nanowires at 1 year

**Fig. 3:**
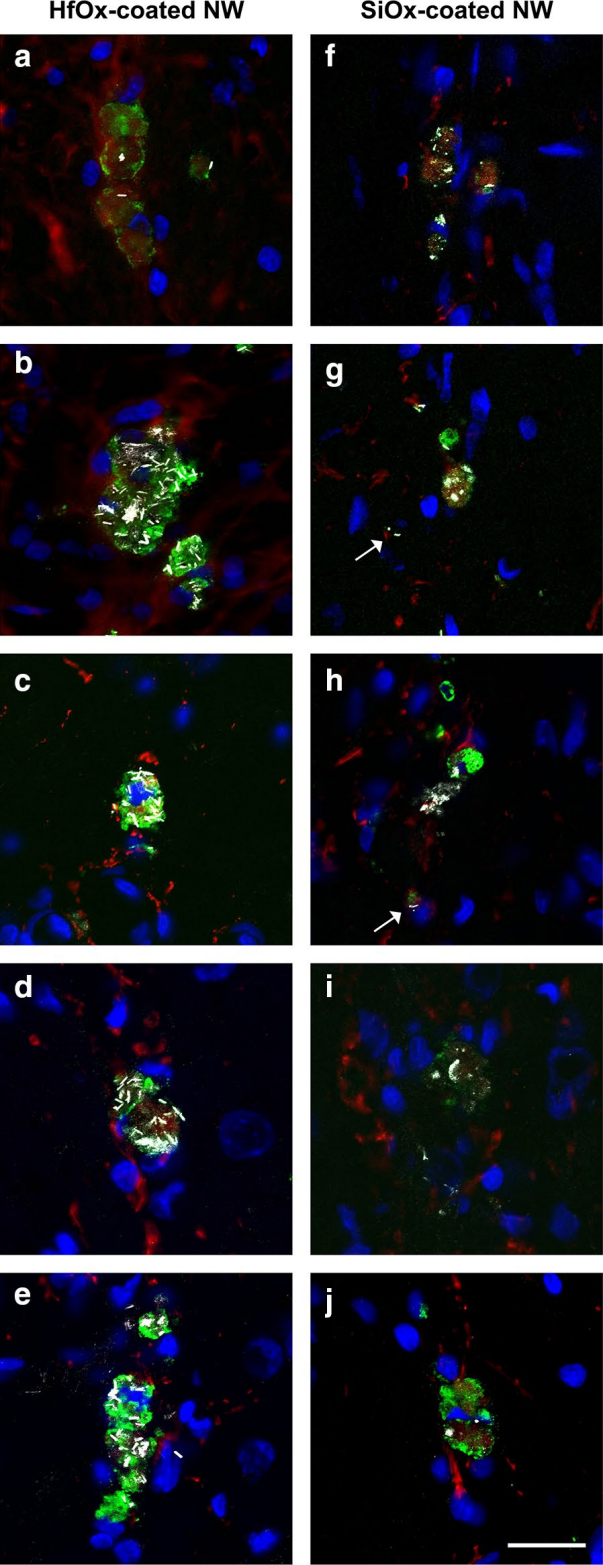
Confocal images 1 year post nanowire injection. Representative laser scanning confocal microscopy images of the scar area after injection of 2 μm long HfOx-coated (**a**–**e**) and SiOx-coated GaP (**f**–**j**) nanowires 1 year post injection. The intact HfOx-coated and fragmented SiOx-coated GaP nanowires are visualized in *white* using scattered laser light mode. The images demonstrate the difference in nanowire cell load and degradability of the two types of nanowires studied. Note also the few rod-shaped SiOx-coated nanowires found in single, small ED1-positive cells (*arrows*). ED1-positive cells (*green*), GFAP-positive cells (*red*), cell nuclei (*blue*) and nanowires (*white*, *scattered laser light*). *Scale bar* 20 μm

Using confocal microscopy (scattered laser light), we found that residual nanowire material from the SiOx-coated GaP nanowires (and occasionally apparently intact nanowires) as well as intact HfOx-coated GaP nanowires engulfed by ED1-positive cells persisted in the tissue (Fig. 3). We could not detect any nanowires or nanowire residues in any other cell types investigated (neuronal cells or astrocytes).

**Fig. 4:**
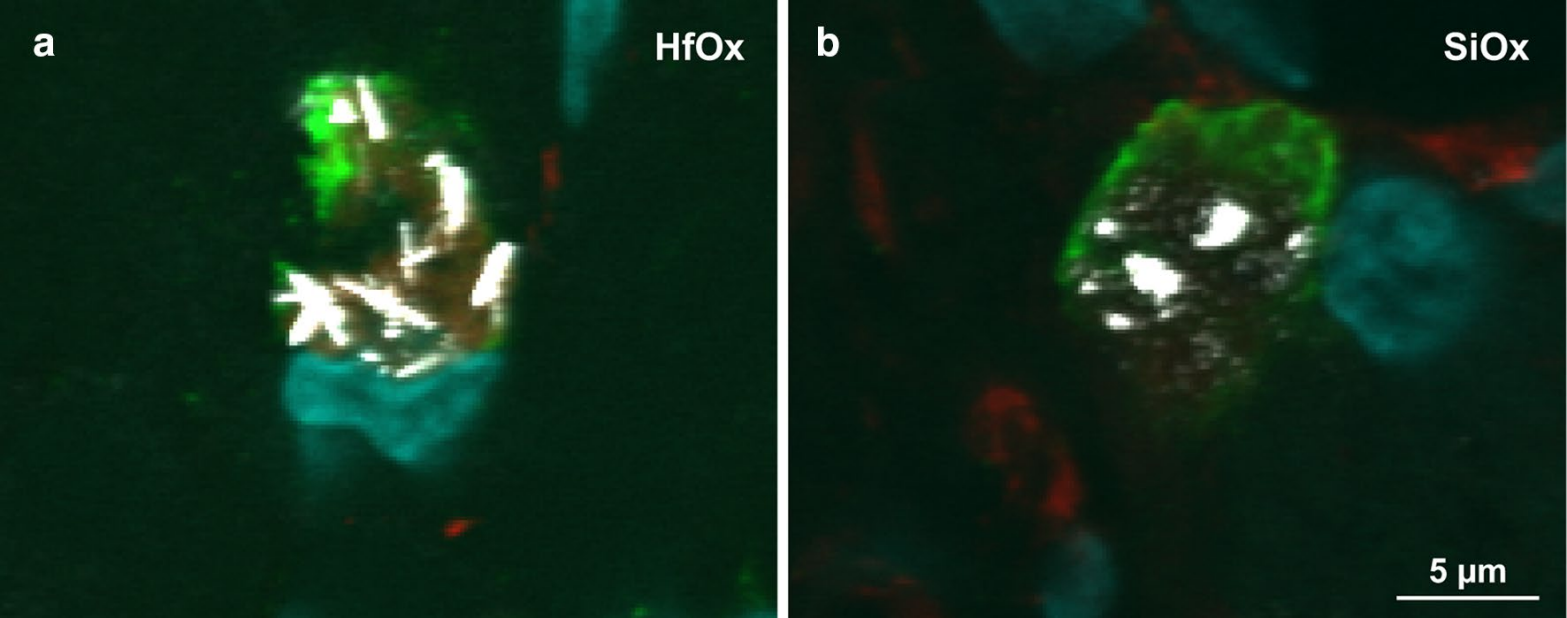
Close-up confocal images. Merged close-up of laser scanning confocal microscopy images showing ED1-positive cells in the scar area after injection of 2 μm long HfOx-coated (**a**) and SiOx-coated GaP (**b**) nanowires 1 year post injection. The images show internalization of intact HfOx-coated and degraded SiOx-coated GaP nanowires in microglia/macrophages. ED1-positive cells (*green*), GFAP-positive cells (*red*), cell nuclei (*blue*) and nanowires (*white*, *scattered laser light*). *Scale bar* 5 µm

Intact HfOx-coated nanowires were found in ED1-positive cells, both in larger cell aggregates as well as in smaller ED1-positive cells (ø 5–10 µm) (Fig. 3a–e). For the degradable SiOx-coated nanowires (Fig. 3f–j), residues from nanowires were also detected inside ED1-positive cells. However, these residues were primarily seen in aggregated hypertrophic ED1-positive cells (ø approximately ≥15 µm), possibly macrophages/microglial cells that have fused to form multinucleated giant cells. SiOx-coated nanowires with an apparently intact shape were also found in the tissue, although, to a lesser extent, and primarily in single, smaller ED1-positive cells (ø 5–10 µm). These images also suggest that less material remains at or in the vicinity of the injection site for the group receiving degradable SiOx-coated GaP nanowires compared to HfOx-coated nanowires (Fig. 3a–j). Figure 4 shows merged close up laser scanning confocal microscopy images of ED1-positive cells containing intact HfOx-coated nanowires (Fig. 4a) or residues from degraded SiOx-coated nanowires (Fig. 4b). The nanowires are visualized in white inside microglia/macrophages using scattered laser light mode.

No nanowires or residues of nanowires were found in any of the cervical lymph nodes investigated.

## Discussion

In this nanosafety study, we evaluated for the first time the long-term (corresponding to half the life time of the animal) impact of degradable nanowires on rat brain tissue and compared to that of non-degradable nanowires. That both the core and the coating of the SiOx-coated GaP nanowires are degraded into fragments were confirmed using confocal microscopy images (Figs. 3, 4). Despite the difference in biostability between SiOx and HfOx coated GaP nanowires, the tissue response and neuronal density were not significantly different. Moreover, both residues from degradable nanowires and intact biostable nanowires remain engulfed in the microglia/macrophages in the brain tissue 1 year post injection, indicating that clearance of nanoparticles from the brain is a very slow process.

The core material of the nanowires used in this study is GaP. GaP is a semiconductor and like many semiconductors, GaP has been shown to be susceptible to corrosion and degradation, a process that leads to a release of Ga ions [28]. We have previously evaluated the in vivo soft tissue inflammatory response after implantation of GaP discs into the abdominal wall of rats [29]. In that study an increase of ED1-positive cells after GaP disc implantation was found both in the reactive capsule and at the disc/tissue interface, possibly reflecting an increased local concentration of toxic Ga ions. In the same study, we also found elevated levels of Ga accumulated in blood, brain, liver and kidneys 12 weeks post implantation, confirming loss of Ga from the implanted GaP discs [29]. GaP is also dissolving in pure physiological saline as well as in hydrogen peroxide (H_2_O_2_, 0.1, 1, 10 %) (to mimic the inflammatory in vivo milieu both inside and outside of activated monocytes/microglia) at 37 °C [29]. These results have been confirmed by Richards et al. [28] using GaP wafers. Taken together, these results demonstrate that the GaP core of the nanowires used in this study degrades both in vitro and in vivo.

In another study we could not detect any sub-acute or neurotoxic effect for the degradable GaP nanowires in the brain 12 weeks after nanowire injection [23]. Furthermore, no gallium was detected in other tissues investigated (blood, kidney and liver) using inductively coupled plasma sector field mass spectrometry (ICP-SFMS). This raised the question whether a possible continuous release of gallium from the nanowires locally in the brain could result in neurotoxicity when the exposure spans over a longer time period. Notably, in the present long-term study, we found no difference in the inflammatory tissue response (i.e. in ED1- or GFAP-positive cell area), in total cell nuclei density or neuronal nuclei density between the degradable and the biostable nanowire group 1 year post injection. Furthermore, we have previously shown that the brain tissue response seen 1 year after an injection of short biostable GaP nanowires is comparable to the brain tissue response seen after vehicle (HBSS only) injection [25]. Taken together, the degradable material does not result in any detectable neurotoxicity 1 year post injection. Note, however, that the amount of Ga introduced in the brain, in our degradable nanowire safety studies, are too low for measuring the amount of released Ga in the brain tissue using spectrographic methods (ICP-SFMS), as in Linsmeier et al. [29].

Feliu and colleagues [30], discuss if a biological environment may impose hostile conditions to nanoparticles, and addresses the question whether inorganic nanoparticles can be designed to be degradable in a controlled manner followed by clearance from the body via renal excretion. We have previously shown that GaP nanowires coated with 20 nm of SiOx were partially degraded after 6 and 12 weeks and that residues were found engulfed by microglia [23]. Furthermore, Hwang et al. [31], demonstrated that silicon-based electronics could be dissolved under physiological conditions. These material dissolving properties are further demonstrated by Peled et al. [32], who reported a dissolution rate of ca. 2.15 nm/day for bare SiOx nanowires (20 nm in diameter) when exposed to PBS (37 °C) leading to non-continuous, segmented, nanowire structures after approximately 7–10 days of exposure. Similar dissolution rates was presented by Zhou et al. [33], who showed that the SiOx nanowires (30 nm in diameter) exhibited dissolution after about 10 days when exposed to PBS or Neurobasal neuron cell culture media (ThermoFisher Scientific, USA) which is a closer analog (than PBS) to an in vivo milieu. These studies further confirm that the GaP nanowire core as well as the SiOx-coating degrades and fragmentizes over time, thus, it might be expected that the SiOx-coated GaP nanowires would be completely dissolved in vivo after 1 year. We therefore hypothesized that the nanowire residues would be fully dissolved and cleared from the brain tissue after 1 year. However, the present data shows that nanowire residues and occasionally intact nanowires persist in the brain, even after 1 year, suggesting that nanoparticle clearance from the brain, if it is taking place, is a very slow process. Indeed, we were not able to detect any nanowires or nanowire residue in the cervical lymph nodes, which is a known destination for emigrating monocytes [34]. This suggests that there is a very small or non-existing migration of microglia/monocytes loaded with degraded or intact nanowires to these lymph nodes, supporting the idea that nanoparticles in the brain might be eliminated in an extremely slow manner.

Our results could be compared to previous reports showing that nanoparticles can accumulate in the brain. For instance, Lee et al. [35], found that small colloidal silver (10 and 25 nm) nanoparticles remain in the brain tissue 4 months after oral delivery. Furthermore, van der Zande et al. [36], found that polyvinylpyrrolidone-coated silver nanoparticles were not cleared from the brain, 84 days post-oral delivery. Kreyling et al. [37], injected radiolabeled polymer-coated gold nanoparticles in the tail vein of rats and were able to measure radioactivity from both the core and shell in the brain already after 1 h. This might suggest that certain nanoparticles can pass the blood brain barrier into the brain and accumulate in the brain since the elimination of the nanoparticles appears to be a more complex process.

When using confocal microscopy to compare activated microglial cells in the injection tract, we found a clear difference in microglial cell load comparing the two nanowire groups (Fig. 3). This suggests that some of the degraded material from the SiOx-coated nanowires have indeed been cleared from the injection tract or that the residue from the nanowires are degraded into very fine fragments, which might not be visualized using confocal microscopy, since smaller debris scatter the light to a lesser degree. While there is an apparent difference in microglia load, there is as mentioned above, no significant difference in the proportion of ED1-positive cell area when comparing the degradable and biostable nanowire groups. It may be speculated that degraded fragments or particles, due to their larger surface area as compared to biostable nanowires, provided an about equal effective stimulus to the microglia cells as that of biostable nanowires.

The degradable nanowires or residues from the degradable nanowires were found only engulfed by ED1-positive cells and were not found inside neurons or astrocytes. Interestingly, we found two phenotypes of activated ED1-positive microglia cells. One phenotype was the hypertrophic ED1-positive cells (ø approximately ≥15 µm) that contained a load of residues from the degradable nanowires or of intact biostable nanowires. These hypertrophic cells were most often found in cell aggregations, possibly multinucleated giant cells. Occasionally, intact biodegradable nanowires were detected in activated single, smaller sized ED1-positive cells (ø 5–10 µm). It is possible, that the presence of nanowires in the brain tissue can give rise to formation of multinucleated giant cells. These are highly activated cells, which produce large amounts of reactive oxygen species [38]. It might then be hypothesized that some of the ED1-positive cells, which engulf nanowires, fuse to become giant cells, which then might accelerate the breakdown of the degradable nanowires into fragments/residues. Hence, the finding that the SiOx-coated nanowires in the single, smaller ED1-positive cells appear to contain more intact nanowires as compared to the large ED1-positive cells or the aggregates of ED1-positive cells is consistent with this hypothesis.

In conclusion, we found engulfed intact nanowires and nanowire residues inside ED1-positive cells only. No obvious bio-safety issues or neurotoxicity were observed after injection of degradable 2 µm long GaP nanowires into rat striatum. Degradable nanowires with apparently intact shape were rarely found in the brain. When degraded, both the coating and the core of the degradable nanowires were fragmented and the remnants were not cleared from the brain even 1 year post nanowire injection. We observed no advantage or disadvantage of using degradable nanowires as compared to biostable nanowires in this long-term nanosafety study. It is important to mention that we can not exclude that other types of degradable materials could offer an advantage over biostable nanobiomaterials in other experimental setups. However, we found that dissolution and removal of inorganic material from the brain are very slow processes; these are very interesting findings which prompt for further studies.

## Additional file

10.1186/s12951-016-0216-7 Excel-file with raw data.
